# Oregano Oil and Harmless Blue Light to Synergistically Inactivate Multidrug-Resistant *Pseudomonas aeruginosa*

**DOI:** 10.3389/fmicb.2022.810746

**Published:** 2022-03-10

**Authors:** Min Lu, Ka Ioi Wong, Xin Li, Fei Wang, Li Wei, Shen Wang, Mei X. Wu

**Affiliations:** ^1^Shanghai Key Laboratory for Prevention and Treatment of Bone and Joint Diseases, Department of Orthopaedics, Ruijin Hospital, Shanghai Institute of Traumatology and Orthopaedics, Shanghai Jiao Tong University School of Medicine, Shanghai, China; ^2^Department of Plastic and Reconstructive Surgery, Shanghai Ninth People’s Hospital, Shanghai Jiao Tong University School of Medicine, Shanghai, China; ^3^Instrumental Analysis Center, Shanghai Jiao Tong University, Shanghai, China; ^4^Department of Dermatology, Wellman Center for Photomedicine, Harvard Medical School, Massachusetts General Hospital, Boston, MA, United States

**Keywords:** blue light, oregano oil, synergistic effects, acute infections, biofilm-associated infections

## Abstract

Blue light (BL) at 405 nm and oregano essential oil (OEO) have shown bactericidal activity by its own. Here, we demonstrated that the two synergistically killed multidrug-resistant (MDR) *Pseudomonas aeruginosa* (*Pa*). *Pa* ATCC19660 and HS0065 planktonic cells and mature biofilms were reduced by more than 7 log_10_ after treatment by BL combined with OEO, in sharp contrast to no significant bacterial reduction with the monotreatment. The duo also sufficiently eliminated acute or biofilm-associated infection of open burn wounds in murine without incurring any harmful events in the skin. The synergic bactericide was attributed mainly to the ability of OEO to magnify cytotoxic reactive oxygen species (ROS) production initiated by BL that excited endogenous tetrapyrrole macrocycles in bacteria while completely sparing the surrounding tissues from the phototoxic action. OEO ingredient analysis in combination with microbial assays identified carvacrol and its isomer thymol to be the major phytochemicals that cooperated with BL executing synergic killing. The finding argues persuasively for valuable references of carvacrol and thymol in assessing and standardizing the bactericidal potential of various OEO products.

## Introduction

*Pseudomonas aeruginosa* (*Pa*) infections have become significant challenges in control of hospital-acquired infections, particularly in patients with severe burns, immunocompromised conditions, and cancers (El Zowalaty et al., 2015; Bassetti et al., 2018). The emergence of multidrug-resistant (MDR) *Pa* results in high rates of mortality either directly or indirectly, since some of these MDR bacteria fail to respond to almost all antibiotics designated to treat them (Obolski et al., 2015). Even last resort of colistin with a relatively low prescription rate confers less and less effectiveness in the control of *Pa* infections owing to increased resistance to the antibiotics (Leeb, 2004; Levy and Marshall, 2004). Apparently, there is an urgent need to research and develop for alternatives in management of MDR *Pa*, especially non-antibiotics and combinatory modalities in fighting against MDR pathogens.

Among non-antibiotic alternatives, essential oils (EOs) have attracted increasing attentions and are considered promising strategies for treating MDR bacteria (Pandey et al., 2017). Natural EOs have been well-known for their enormous potentials as microbicides in ancient China and Egypt. We previously investigated antimicrobial activities of 42 EOs extracted from Chinese endemic aromatic herbs and spices against a panel of bacteria and fungi (Lu et al., 2013a,b,c). Among these EOs tested, 11 of them showed a superior antimicrobial activity against clinical and agricultural pathogens (Lu et al., 2013a,b,c). For instance, oregano essential oil (OEO) safely and substantially reduced bacterial loads after topical application onto the infected murine burns without development of any resistance to the treatment even after repeated treatments (Lu et al., 2018).

Another innovative sterile technique is antimicrobial blue light (BL) and has been extensively studied for the treatments of MDR microbes (Wang et al., 2016, 2019). The mechanism underlying BL-mediated bactericide is proven to specifically stimulate the excessive production of reactive oxygen species (ROS) by its ability to excite endogenous non-metallated tetrapyrrole macrocycles that are produced abundantly within most of bacteria but not in mammalian cells (Surdel et al., 2017; Wang et al., 2017). BL confers significant advantages as an alternative to antibiotics due to its faster action and great effectiveness irrespective of antibiotic susceptibility (Wang et al., 2017).

BL and EOs both possess multi-target features, i.e., targeting different organelles of bacterial cells, which would greatly shield the development of resistance to the modalities. In view of this, we investigated the bactericidal efficacy by combining BL and OEO against a standard strain of *Pa* ATCC19660 and a clinical isolate of *Pa* HS0065 *in vitro* and *in vivo*. We found that a combination of OEO with BL substantially and safely reduced bacterial loads in acute and biofilm-associated burn infections as compared to either alone, which was mainly attributed to its key ingredients carvacrol and its isomer thymol. This synergistic and non-antibiotic approach holds great promise to effectively and safely manage pathogenic *Pa* infections.

## Materials and Methods

### Phytochemicals, Light Source, *Pseudomonas aeruginosa* Strains, and Human Cells

OEO and seven phytochemicals (>98% purity) were bought from Sigma-Aldrich. A light-emitting diode (LED) of BL, which had a peak width of 405 ± 12.5 nm, and a soft white LED bulb (3W, A15) were purchased from Thorlabs and General Electric, respectively. The irradiation was fixed to 55 mW/cm^2^ for all experiments and measured on a PM100D power/energy meter (Thorlabs). *Pa* strains used in this study were verified MDR *via* conventional microbiology tests. *Pa* HS0065 was isolated from a burn-infected patient in Huashan Hospital in Shanghai, and a luminescent *Pa* ATCC19660 was employed for monitoring infection in alive mice by a Lumina *in vivo* image system (IVIS) (PerkinElmer) (Dai et al., 2013b). The human fibroblasts (ATCC PCS-201-012) were cultured for 2–3 days at 37°C, 5% CO_2_ in Dulbecco’s modified Eagle’s medium (DMEM) containing 10% fatal bovine serum (FBS), 100 U/ml penicillin, and 100 μg/ml streptomycin.

### Oregano Essential Oil Components Identified by Gas Chromatography/Mass Spectrometry

OEO constituents were analyzed by a gas chromatography (GC) (Agilent 6980) coupled to mass spectrometry (MS) (Agilent 5973N) *via* a fused-silica capillary column (HP-5MS: 30 m × 0.25 mm i.d.). The operating procedures were as follows: initial oven temperature, 60°C for 10 min; gradually increased to 220°C at 3°C/min; maintained at 220°C for additional 10 min; then elevated to 240°C with an increment of 1°C/min. The temperature of the injector port and ionization source was 250°C. The carrier gas was helium at 0.8 ml/min. Ionization voltage of MS in the EI mode was 70 eV. OEO constituents were identified and confirmed by comparing the retention index (RI) in reference to n-alkanes and the mass spectra of authentic compounds in NIST, Wiley databases, and Adams library.

### Synergistic Effects of Blue Light and Oregano Essential Oil/Compounds Against *Pseudomonas aeruginosa* Planktonic Cells

OEO or seven compounds each were dissolved at 40 mg/ml in N,N-dimethylformamide (DMF) as stock solutions. To assess synergistic effects, 2,970 μl of *Pa* planktonic cells in phosphate-buffered saline (PBS) at 7.0 log CFU/ml was mixed with 30 μl of OEO or an indicated compound at varying concentrations in a 35-mm Petri dish and then irradiated immediately with BL at 55 mW/cm^2^ while stirring at 60 rpm. Before or at indicated times after treatments, 50 μl aliquot was collected and assayed on brain heart infusion (BHI) agar plates to determine colony-forming units (CFU) (Jett et al., 1997). For comparisons, BL alone or OEO/compounds alone was also assayed in parallel. The synergistic degrees between BL and OEO/compounds against the two *Pa* strains were determined by *S*-values obtained through the following formula or the Bliss independence model:

*S*-values = (logCFU/ml_BL_/logCFU/ml_Control_) (logCFU/ml_OEO(or carvacrol or thymol)_/logCFU/ml_Control_) − (logCFU/ml_BL+OEO (or carvacrol or thymol)_/ logCFU/ml_Control_) (Courtney et al., 2017; Lu et al., 2021b). LogCFU/ml_BL_, logCFU/ml_OEO (or carvacrol or thymol)_, logCFU/ml_BL+OEO (or carvacrol or thymol)_, and logCFU/ml_Control_ numbers were viable bacteria left after an indicated treatment.

### Verification of Any Possible Resistance to the Combined Therapy

The two *Pa* strains were evaluated for possible resistance to the combined therapy by repeated treatments per a published protocol with some modifications (Lu et al., 2021b). In brief, *Pa* suspensions were exposed severally to a sublethal dose of the combined therapy, which could inactivate 3 log CFU/ml of bacterial growth. The initial sublethal dose was 60 J/cm^2^ BL combined with 0.2 mg/ml OEO for *Pa* ATCC 19660 or 30 J/cm^2^ BL and 0.2 mg/ml OEO for *Pa* HS0065. After first treatment cycle, bacteria that survived the treatment were re-cultured for the next suppressive growth cycle with a newly defined sublethal dose of the combined therapy, if necessary. The procedure was repeated for 20 successive cycles. Resistance was estimated by any significant rise in sublethal doses or CFU with increasing passages up to 20 cycles.

For comparison, ampicillin (AMP) resistance was assessed in parallel. Briefly, *Pa* suspension was added into 96-well plate at 200 μl/well containing serial dilutions of AMP as described (de Breij et al., 2018). After 24 h incubation, the lowest AMP concentration that could completely inactivate bacterial growth was defined as a minimum inhibitory concentration (MIC). Then, 10 μl of *Pa* suspension grown in BHI broth containing 1/2 MIC AMP was added into a fresh BHI broth. The bacteria were cultured and determined for its MIC again as above. AMP resistance was determined if any significant rise in MIC was found within 20 consecutive passages.

### Synergistic Effects of Blue Light and Oregano Essential Oil/Compounds Against *Pseudomonas aeruginosa* Mature Biofilms

*Pa* grown in trypticase soy broth (TSB) was transferred into a 96-well plate at 100 μl/well and cultured for 3 days to establish biofilms. The biofilms were washed twice with PBS, soaked in 200 μl of OEO, carvacrol, or thymol each at 0.8 mg/ml, and immediately illuminated with BL at 100 J/cm^2^. Antibacterial efficacy of BL alone at 100 J/cm^2^ or OEO, carvacrol, or thymol alone each at 0.8 mg/ml against the biofilms was assayed side-by-side as controls. Bacteria left in the biofilms were enumerated *via* CFU assay after dislodging them in 200 μl PBS by 5-min ultrasonic vibration as described (Jett et al., 1997). The synergy between BL and OEO/compound (carvacrol or thymol) against the two *Pa* mature biofilms was verified by *S*-values calculated with the Bliss Independence model above (Courtney et al., 2017; Lu et al., 2021b).

### Therapy of Acute Burn Infections

All animal studies were approved by the Shanghai Jiao Tong University Animal Study Committee. The lower dorsal skin of 8-week-old BALB/C mice were hair removed. A brass block at a size of 1 cm^2^ was heated in 100°C boiling water before being applied onto the hairless skin for 5–8 s so that a full-thickness third-degree burn could be generated. For acute burn infection, approximately 6.5 log CFU luminescent *Pa* ATCC19660 in 50 μl PBS were inoculated, after 10 min of the burn, onto the burns for 30 min. The infected burns were spread uniformly with 50 μl OEO (10 mg/ml) prior to BL exposure for varying lengths. Likewise, the antibacterial activity was determined with BL, OEO, or PBS combined with sham light as controls. Bacterial luminescence images in burns were acquired by a Lumina IVIS (PerkinElmer) (Lu et al., 2021b). Bacterial luminescence intensity was measured with the Living Image software (Xenogen). All mice were sacrificed on day 8 after bacterial inoculation to determine viable bacteria remaining on wounds or in blood. Serial dilutions of wound homogenates and blood were cultivated on BHI agar plates with the supplements of Skirrow for bacterial enumeration.

### Management of Biofilm-Associated Burns

Full-thickness murine burns were created as above and covered evenly with 50 μl of *Pa* HS0065 containing 7.5 log CFU in PBS (Wang et al., 2016), 3 days after which 20 μl of OEO at 20 mg/ml was distributed to the wounds, along with 60 or 120 J/cm^2^ BL irradiation. The therapeutic effect of OEO, BL irradiation, or PBS together with sham light (control) was also evaluated simultaneously as controls. On day 15 after various treatments, all mice were euthanized and perfused with PBS through the heart. The wounds and vital organs such as lungs, livers, and spleens were dissected and processed by a homogenizer in 2 ml of PBS for bacterial CFU determination as above (Jett et al., 1997).

### Measurements of H_2_O_2_ and HO in the Absence or Presence of Bacteria or Protoporphyrin IX

OEO, carvacrol, or thymol solution each at 0.1 mg/ml with or without 10^7^ CFU/ml of *Pa* HS0065 was aliquoted to a 35-mm Petri dish at 3 ml/dish and then exposed to 30 J/cm^2^ BL or sham light. After the treatment, 50 μl solution was collected from each dish, mixed with an equal amount of Amplex Red reagent/HRP working solution, and reacted at room temperature for 20 min. The amount of H_2_O_2_ was estimated by a microplate spectrophotometer at an excitation/emission wavelength of 571/585 nm (Zhao et al., 2012). To measure ⋅HO production, the solution was collected similarly and mixed for 15 min with the molecular probe 3′-(p-hydroxyphenyl) fluorescein (HPF) at a final concentration of 5 μM. A yield of HO was quantified by a microplate spectrophotometer at excitation/emission wavelength of 505/525 nm (Morones-Ramirez et al., 2013). Similar measurements of H_2_O_2_ and ⋅HO production were also carried out in the presence of 10 μM protoporphyrin IX (PPIX) in place of bacteria whereby PPIX provided an exogenous photosensitizer to simulate photo-chemical reaction in bacteria.

### Cell Viability

Human fibroblasts were treated by 0, 0.1, 0.2, 0.4, or 0.8 mg/ml OEO along with 100 J/cm^2^ BL. Cell viability was estimated with CCK-8 kit (Beyotime) based on the absorbance values at 450 nm. Moreover, human fibroblasts and bacteria were mixed and cultured together at an 1:10 ratio, and the co-culture was exposed to a lethal dose of the duo (50 J/cm^2^ BL and 0.6 mg/ml of OEO). Then, 10 μM calcein-AM and propidium iodide (PI) solutions were added to the co-cultures to mark viable cells and dead cells, respectively, according to the manufacturer’s instruction. The images of cells were captured by the green/red fluorescence channel in the FluoView FV1000-MPE confocal microscope (Olympus).

### Toxicity Assessment of the Combinatory Treatment *in vivo*

Mice were anesthetized, hair removed on the lower dorsal skin, and treated by 50 mg/ml of OEO (50 μl) alongside 150 J/cm^2^ BL irradiation as above. The treatment was repeated daily for consecutive 3 days. Blood samples, skin biopsies, and vital organs of liver and kidney were taken 24 h after the final treatment. The index of alanine aminotransferase (ALT), aspartate aminotransferase (AST), alkaline phosphatase (ALP), creatinine (Cr), and blood urea nitrogen (BUN) were measured by automatic analytical instruments (Abbott AxSYM) through standard methods. The skin, liver, and kidney biopsies were immersed in 10% phosphate-buffered formalin to fix the tissue and processed for hematoxylin and eosin (H&E) staining for histological observation and visualization in Nanozoomer 2.0 HT (Hamamatsu). The resultant images were analyzed by using NDP viewer software (Hamamatsu). Possible DNA damage for skin biopsies was evaluated by the DeadEnd Fluorometric TUNEL staining per the manufacturer’s instructions (Promega). DNA fragmentation was also induced in some tissue sections with 10 U/ml RQ1 RNase-free DNase I as positive controls. Fluorescence images were captured in a FluoView FV1000-MPE confocal microscope (Olympus).

### Statistical Analyses

All data are mean ± standard deviations (SDs). Two tailed Student’s *t*-test or one-way ANOVA analysis was performed to determine statistical significance between two groups or multiple groups. The survival curves were compared by Kaplan–Meier method. GraphPad Prism 7.0 (GraphPad software) was used for all statistical analyses, and *p* < 0.05 indicated statistically significant.

## Results

### The Antibacterial Activity of Oregano Essential Oil and Its Synergy With Blue Light Are Ascribed Primarily to Carvacrol and Thymol

**FIGURE 1:**
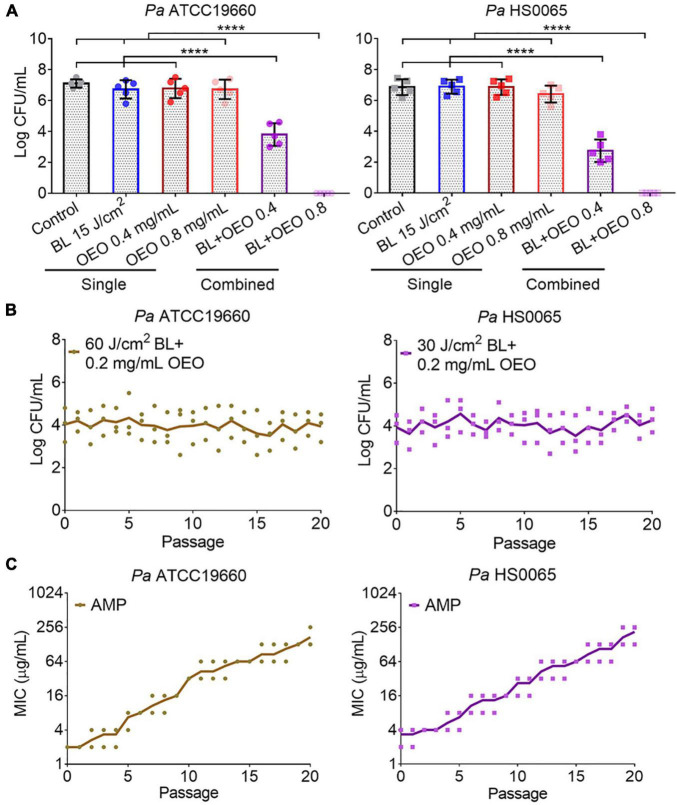
Synergistic bactericidal activity of BL and OEO and no resistance development. **(A)** Synergy between OEO and BL. OEO was evaluated for its antibacterial activity at 0.8 or 0.4 mg/ml against planktonic *Pa* ATCC19660 (left) and *Pa* HS0065 (right), respectively, in the presence or absence of 15 J/cm^2^ BL. The bacteria were sham treated (control) or treated with BL at 15 J/cm^2^ alone as controls. **(B)** Resistance of *Pa* ATCC19660 (left) and *Pa* HS0065 (right) to 60 and 30 J/cm^2^ BL combined with 0.2 mg/ml OEO, respectively. **(C)** Resistance of the two bacterial strains to ampicillin (AMP). Data represent the result of two independent experiments each performed in triplicate (*n* = 6) **(A)** or triplicate assays **(B,C)** ****p < 0.0001; and ns, no significance.

OEO was found to kill a standard strain *Pa* ATCC19660 and a clinical isolate *Pa* HS0065 in planktonic cultures equivalently with an MIC of 0.8 mg/ml, i.e., the minimal concentration of OEO needed to completely suppress the growth of 10^7^ CFU/ml of the bacteria after 24 h treatment in a standard MIC assay (Table 1). Strikingly, the same concentration of OEO exterminated 7.0 log CFU/ml of *Pa* ATCC19660 (left panel) or *Pa* HS0065 (right panel) in 5 min, rather than 24 h, should OEO be combined with 15 J/cm^2^ of BL (Figure 1A). The quick and efficacious bactericidal action of the duo therapy was unparalleled to any of monotherapies (Figure 1A). As can be seen in Figure 1A, 15 J/cm^2^ of BL alone or 0.8 mg/ml OEO alone failed to significantly suppress growth of the bacteria under similar conditions. Reduction in the OEO concentration by half (0.4 mg/ml) could still eliminate 2.9 CFU/ml of *Pa* ATCC19660 or 3.8 CFU/ml of *Pa* HS0065 in the presence of 15 J/cm^2^ of BL, highly significant compared with either BL or OEO alone (*p* < 0.0001) (Figure 1A). We next assessed whether *Pa* ATCC19660 and *Pa* HS0065 were susceptible to development of resistance to the duo therapy as previously described (Lu et al., 2021b). The two bacterial strains appeared not to develop any resistance to the combinatory therapy after 20 consecutive cycles of sublethal treatments (Figure 1B). In contrast, the MICs of *Pa* ATCC 19660 and *Pa* HS0065 to ampicillin (AMP) arose by 50- and 40-fold, respectively, after 20 passages (Figure 1C).

**TABLE 1:** Bactericidal activity and synergy with BL of OEO or its major ingredients.

No	Constituents	RI^a^	RI^b^	Peak area (%)^c^	*Pa* ATCC 19660	*Pa* HS0065	*Pa* ATCC 19660				*Pa* HS0065			
							BL 15J/cm^2^	Com 0.4 mg/ml	BL + Com	*S*-value	BL 15 J/cm^2^	Com 0.4 mg/ml	BL + Com	*S*-value
					**MIC (mg/mL)**		**Log reduction of CFU/mL**				**Log reduction of CFU/mL**			
1	α-thujene	926	924	0.25										
2	**α *-pinene***	***939***	***932***	***1.42***	***>1***	***>1***	***0***	***0***	***0^ns^***	***0***	***0***	***0***	***0^ns^***	***0***
3	Camphene	953	946	0.17										
4	1-octen-3-ol	982	974	0.14										
5	***Myrcene***	***991***	***988***	***2.23***	***>1***	***>1***	***0***	***0***	***0^ns^***	***0***	***0***	***0***	***0^ns^***	***0***
6	α-phellandrene	1,002	1,002	0.82										
7	α-terpinene	1,018	1,014	0.71										
8	***p-cymene***	***1,026***	***1,020***	***4.82***	***>1***	***>1***	***0***	***0***	***0^ns^***	***0***	***0***	***0***	***0^ns^***	***0***
9	Limonene	1031	1024	0.27										
10	**γ *-terpinene***	***1,060***	***1,054***	***3.89***	***1***	***1***	***0***	***0***	***0^ns^***	***0***	***0***	***0***	***0^ns^***	***0***
11	Terpinolene	1,092	1,086	0.21										
12	***Linalool***	***1,100***	***1,095***	***1.38***	***1***	***1***	***0***	***0***	***0^ns^***	***0***	***0***	***0***	***0^ns^***	***0***
13	Borneol	1,165	1,165	0.32										
14	Terpinen-4-ol	1,178	1,174	0.51										
15	***Thymol***	***1,295***	***1,289***	***7.02***	***0.8***	***0.8***	***0***	***0***	***3.2*** ***	***0.46***	***0***	***0***	***1.5*** *	***0.21***
16	***Carvacrol***	***1,315***	***1,298***	***71.93***	***0.8***	***0.8***	***0***	***0***	***4.5*** ****	***0.64***	***0***	***0***	***4.9*** ****	***0.70***
17	Caryophyllene	1,426	1,417	0.82										
18	α-humulene	1,456	1,452	0.27										
19	β-bisabolene	1,509	1,505	0.35										
20	Isocaryophyllene oxide	1,585	1,582	0.76										
	Total			98.29										
	***OEO***				***0.8***	***0.8***	***0***	***0***	***2.9*** ***	***0.41***	***0***	***0***	***3.8*** ****	***0.54***

*The chemical compounds (Com) of OEO were identified by GC-MS. Seven main compounds > 1% of peak area were marked in bold and their MIC values evaluated against Pa ATCC19660 and Pa HS0065, alongside OEO. The antibacterial activities of BL alone at 15 J/cm^2^, OEO/main compounds each at 0.4 mg/ml, or when combined with BL were assessed against Pa ATCC19660 and Pa HS0065.*

*^a^Retention index (RI) was calculated based on the C6–C28 n-alkanes reserved on the HP-5MS capillary column.*

*^b^Retention index (RI) was obtained from Kovat’s retention index.*

*^c^Expressed as percentage of the total peak area of unadjusted chromatograms.*

*Synergistic bactericidal activities between BL and OEO, carvacrol or thymol were marked in red. 0 < S-value < 1 implicates that synergic effect happened; in the meantime, S-value < 0 implicates antagonistic interaction between BL and OEO/compound.*

(****p < 0.0001; ***p < 0.001; *p < 0.05; and ns, no significance.

To determine specific ingredients responsible for the bactericidal activity and its synergy with BL, chemical constituents of OEO were analyzed by GC-MS. Twenty compounds were accounted for 98.29% of the total ingredients of OEO, among which a monoterpenoid phenol carvacrol and its isomer thymol along with α-pinene, myrcene, p-cymene, γ-terpinene, and linalool were the predominant ingredients of OEO (Table 1). Interestingly, among the seven compounds tested, thymol, and carvacrol showed the most sterilization activity with the same MIC as OEO (0.8 mg/ml), while other five compounds all had an MIC ≥ 1 mg/ml for both *Pa* strains (Table 1). The results suggest that thymol and carvacrol are the primary ingredients responsible for the bactericidal activity of OEO. Similar to OEO, carvacrol or thymol at 0.4 mg/ml did not exhibit significant bactericidal activity by its own but eliminated 4.5 or 3.2 log CFU/ml of *Pa* ATCC19660 and 4.9 or 1.5 log CFU/ml of *Pa* HS0065, respectively, when combined with 15 J/cm^2^ BL (Table 1). Notably, the bactericidal activity mediated by carvacrol and BL was greater than that of OEO plus BL for both strains. In contrast to carvacrol and thymol, other five ingredients, namely, α-pinene, myrcene, p-cymene, γ-terpinene, and linalool, did not show any enhanced bactericidal activity when paired with BL under similar conditions (Table 1). The study concludes that active bactericidal ingredients of OEO, especially its synergy with BL, are composed primarily of carvacrol and thymol.

### Blue Light and Oregano Essential Oil/Compounds Synergically Killed *Pseudomonas aeruginosa* Planktonic Cells and Mature Biofilms

**FIGURE 2:**
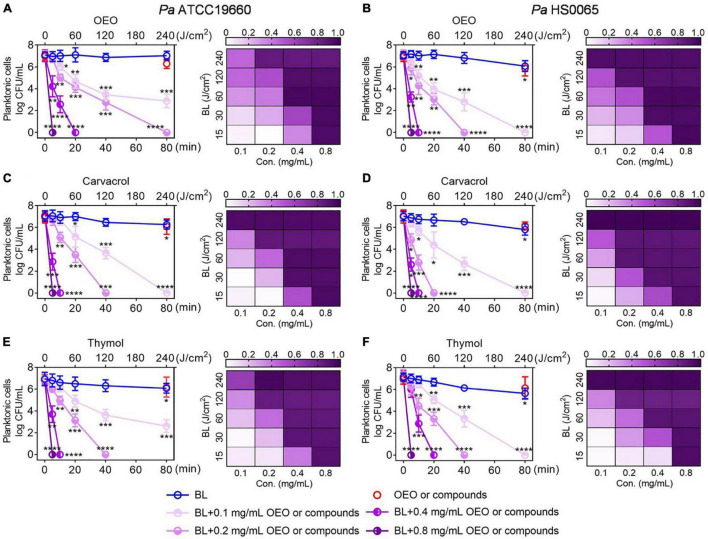
Combination of BL with OEO **(A,B)**, carvacrol **(C,D)**, or thymol **(E,F)** synergistically inactivates *Pa* planktonic cells. *Pa* ATCC19660 (left) and *Pa* HS0065 (right) were treated with increasing lengths of BL irradiation in combination with indicated concentrations of OEO, carvacrol, or thymol. Killing curves (left) were shown on the left, and the corresponding checkerboards were on the right in which S-values (0–1.0) for indicated treatments were assessed by the Bliss Independence model as detailed in section “Materials and Methods”. Results are expressed as mean ± SD of three independent experiments each run in triplicate. ****p < 0.0001; ***p < 0.001; **p < 0.01; and *p < 0.05.

The bactericidal effect mediated by BL combined with carvacrol or thymol resembled that of OEO exhibiting synergism in both BL- and phytochemical-dose-dependent fashions. As shown in Figure 2, with an increasing length of BL irradiation from 0 to 80 min, which corresponded to a fluence rise from 0 to 240 J/cm^2^, 7.0 log CFU/ml of *Pa* ATCC19660 (left panel) or *Pa* HS0065 (right panel) was completely exterminated after 4 min BL irradiation in the presence of 1 × MIC or 0.8 mg/ml of carvacrol (middle) or thymol (bottom), comparable to that of OEO (upper). Similar bactericidal effectiveness was also obtained by reducing the concentration of OEO, carvacrol, or thymol to 0.2 or 0.4 mg/ml from 0.8 mg/ml, while extending the irradiation time to 80 or 20 min, respectively (Figure 2). The inversed doses between the two varied with bacterial strains and individual compounds with *Pa* HS0065 (right) more sensitive than *Pa* ATCC19660 (left) and carvacrol more potent than OEO or thymol for both pathogens (Figure 2). It should be emphasized that the killing was not ascribed to heat because BL irradiation for 80 min alone or at a fluence as high as 240 J/cm^2^ killed < 0.5 log CFU/ml of *Pa* ATCC19660 or 1.0 log CFU/ml of *Pa* HS0065 (Figure 2). The synergy of the combined therapies was verified by the Bliss Independence model, and synergistic degrees were established by *S*-values in the corresponding checkerboards on right (Figure 2); 0 < *S*-value < 1 indicates synergy, while *S*-value < 0 indicates antagonism. Apparently, the synergy was strengthened with an increasing BL intensity or a rising concentration of OEO, carvacrol, or thymol. The strongest synergy was seen at the upper right corner and the lowest at the lower left corner of the checkerboards.

**FIGURE 3:**
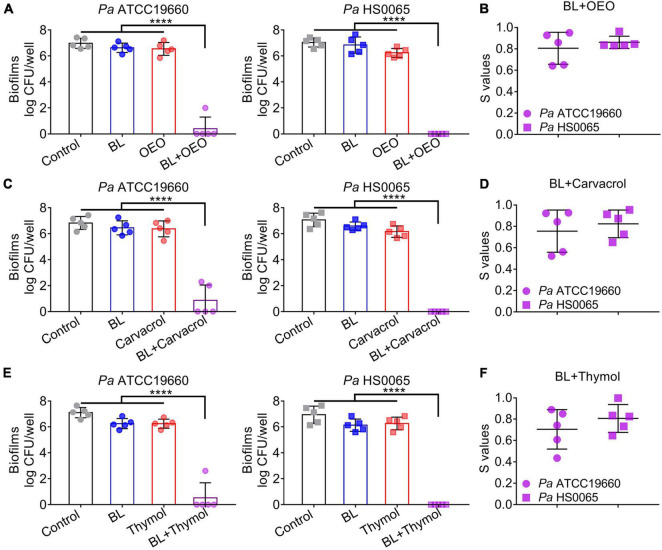
A combination of BL and OEO (upper), carvacrol (middle), or thymol (bottom) synergistically eradicates Pa mature biofilms. **(A,C,E)** Anti-biofilm efficacies of BL at 100 J/cm^2^, OEO/compounds each at 0.8 mg/ml, or both against established biofilms formed by *Pa* ATCC19660 (left) and *Pa* HS0065 (right). **(B,D,F)** Synergistic degrees (*S*-values) of the combinatory treatment for killing *Pa* biofilms were evaluated by Bliss Independence model as Figure 2. Data are presented as mean ± *SD* of five independent experiments each run in triplicate. ****p < 0.0001.

We then extended the treatment to 3-day-old mature biofilms formed by *Pa* ATCC19660 and *Pa* HS0065. A combination of BL at 100 J/cm^2^ and OEO/compounds each at 0.8 mg/ml efficaciously reduced 7 log CFU per well, whereas only < 0.5 log CFU per well were eliminated by the monotherapy under similar conditions (Figures 3A,C,E). The synergy in eradication of *Pa* biofilms between BL and OEO/compounds was once again proven by the Bliss Independence model (Figures 3B,D,F). All *S*-values were > 0.7, on average, indicating excellent synergism of the combined therapy against *Pa* mature biofilms.

### Antibacterial Efficacy of Blue Light Paired With Oregano Essential Oil in the Treatment of Acute and Biofilm-Inflicted Wounds

**FIGURE 4:**
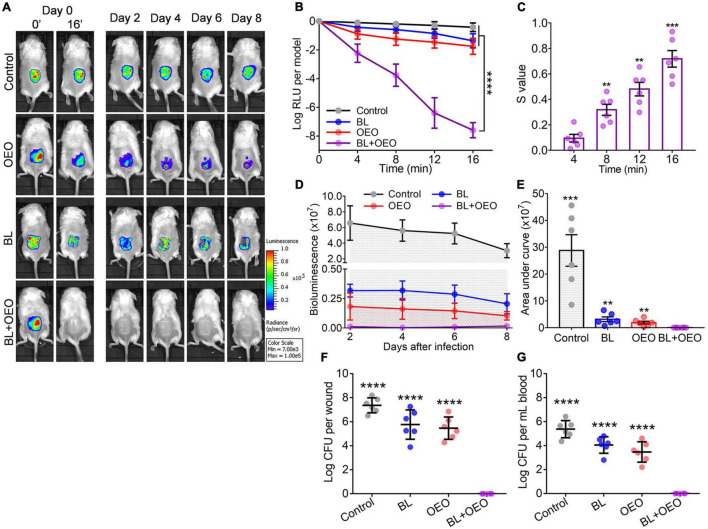
Efficacies of BL plus OEO in the local treatment of burn wounds acutely infected with *Pa* ATCC19660. Murine third-degree burns were acutely infected with 6.5 log CFU of bioluminescent *Pa* ATCC19660 in 50 μl for 30 min. The wounds were subjected to treatment of PBS alongside sham light (control), an indicated dose of BL, 50 μl of OEO at 10 mg/ml, or both. **(A)** Luminescent images of the representative wounds were obtained at specific times. **(B)** Averaged logarithmic relative luminescence units (log RLU) were attained to define the bactericidal efficacy of various treatments. **(C)** Synergistic degrees between BL and OEO were assessed as Figure 2. **(D)** Mean bacterial luminescence of murine burns were real-time recorded at days 2, 4, 6, and 8 after the aforementioned treatments. **(E)** Average areas under the bacterial luminescent curve are shown in **(D)**, which encompasses an entire bacterial load over an 8-day infectious period. **(F,G)** The bacterial load was examined on wound **(F)** and in blood **(G)** on day 8 of the infection. The minimal detection of the infection is 40 CFU on the wound and 20 CFU/ml in blood. Data are presented as mean ± *SD* of six biological replicates. ****p < 0.0001; ***p < 0.001; and **p < 0.01.

The third-degree full thickness burns were infected with luminescent *Pa* ATCC19660 for 30 min as acute infection model. The wounds were treated with sham (control), monotherapies, or duo therapy (BL + OEO) (Figure 4). Bacterial luminescence on the wounds vanished 16 min after 50 J/cm^2^ BL and OEO treatment and did not recrudesce within 8 days (Figure 4A). On the contrary, the bioluminescent signal remained unaltered in the controls, while there was only a limited reduction in the animals receiving monotherapy over 8 days (Figure 4A). On average, 7.6 log RLU per model was exterminated directly by the combined therapy, whereas only < 2.0 log RLU per model was eliminated following the monotherapy (Figure 4B), a representative of more than 5.0 log more efficient killing of the combined therapy over the monotherapy (Figure 4B; *p* < 0.0001). The synergistic effect of the duo therapy was ascertained with the Bliss Independence model in a BL-dose-dependent manner (Figure 4C).

The bacterial luminescence on the wounds was also tracked every other day till day 8 after individual therapies (Figure 4D). No bacterial regrowth was found at the wounds in mice treated with the combined therapy (Figures 4D,E). On day 8 of the experiment, all mice were sacrificed to examine bacteria loads on wounds (Figure 4F) or in blood (Figure 4G). Only in the group receiving the combinatory therapy were bacteria detected neither on the wound nor in blood, in sharp contrast to a high CFU number in both wound and blood in mice sham treated or treated by a monotherapy. The results underscore the importance of effective controlling skin wound infections in the prevention of systemic infection that could cause sepsis or death.

**FIGURE 5:**
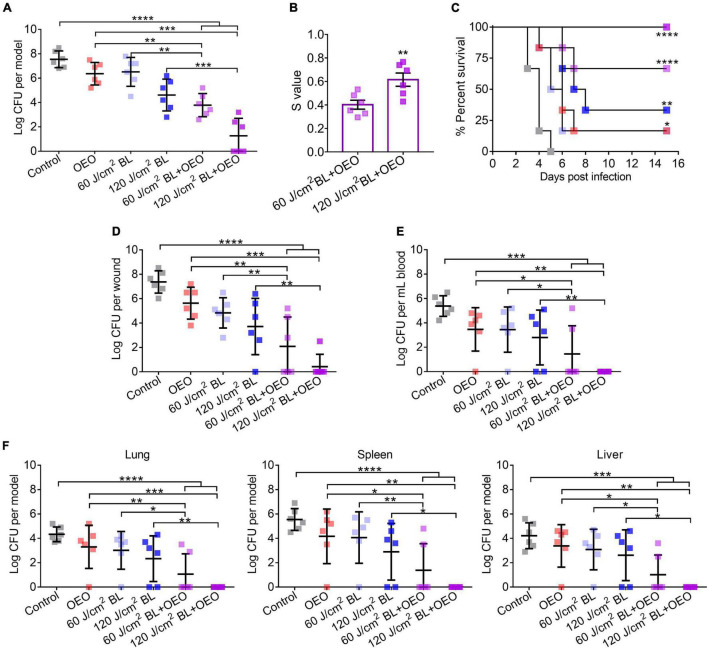
Efficacies of BL and OEO in prevention of fatal biofilm-associated burn infections. The third-degree burns were infected with 7.5 log CFU of *Pa* HS0065. Three days after the infection, the wounds were exposed to sham (control), 50 μl of OEO at 20 mg/ml alone, BL alone at a dose of 60 or 120 J/cm^2^, or both, respectively. **(A)** The infection was treated by indicated treatments. **(B)** Synergistic degrees between BL (60 or 120 J/cm^2^) and OEO for biofilm damage were established as Figure 2. **(C)** Kaplan–Meier survival curves of biofilm-associated infection following various treatments. **(D–F)** Mice were sacrificed on day 15 after treatments to determine the bacterial loads on wounds **(D)** and in blood **(E)**. In addition, bacterial burdens in the vital organs of the lung, spleen, and liver were quantified after dissection and homogenate **(F)**. Data are expressed as mean ± *SD* from six biological replicates. Detection limitations are 40 CFU for wounds or organs and 20 CFU/ml for blood. ****p < 0.0001; ***p < 0.001; **p < 0.01; *p < 0.05.

To grow biofilms on wounds, 7.5 log CFU of *Pa* HS0065 was inoculated onto the third-degree burns and incubated for 3 days. The burns were treated with sham, 20 mg/ml of OEO in 50 μl PBS alone, 60 or 120 J/cm^2^ of BL alone, or both, respectively (Figure 5). On day 1 after an indicated treatment, 50% of mice receiving the combined 120 J/cm^2^ BL and OEO treatment gave rise to a sterile wound (Figure 5A). The synergy between OEO and BL became stronger as BL or OEO increased (Figures 5A,B). A fluence of 60 or 120 J/cm^2^ BL in the presence of OEO both prevented the mice from death significantly during a course of 15-day infection, with 67 and 100% survival rates, respectively. On the contrary, the survival rates were much lower, only 0, 17, 17, and 34% in sham or monotherapy of OEO, 60 J/cm^2^ BL, and 120 J/cm^2^ BL, respectively (Figure 5C).

On day 15 after various treatments, all mice were sacrificed to determine the bacterial loads in wounds (Figure 5D), blood (Figure 5E), and vital organs (Figure 5F) of the lung, spleen, and liver. As expected, the combined therapy showed the most potent therapeutic effect than any of the monotherapies. Especially after treatment of 120 J/cm^2^ BL combined with OEO, the percentages of mice with a sterile wound, blood, lung, spleen, and liver were 83, 100, 100, 100, and 100%, respectively (Figures 5D–F). The results indicate that the combined therapy effectively prevents system infection and holds back the bacteria from wound into the blood. Compared with *in vitro* experiment (Figure 3A), the bactericidal effect *in vivo* might be contributing to two reasons, first the different growth phases to bacteria and second the innate immune response that was aroused to increase phagocytosis or oxidative, which led to more effective treatment although using similar dose of BL.

### Intracellular Reactive Oxygen Species Generated Exclusively in Bacteria Are Responsible for Bactericidal Action

**FIGURE 6:**
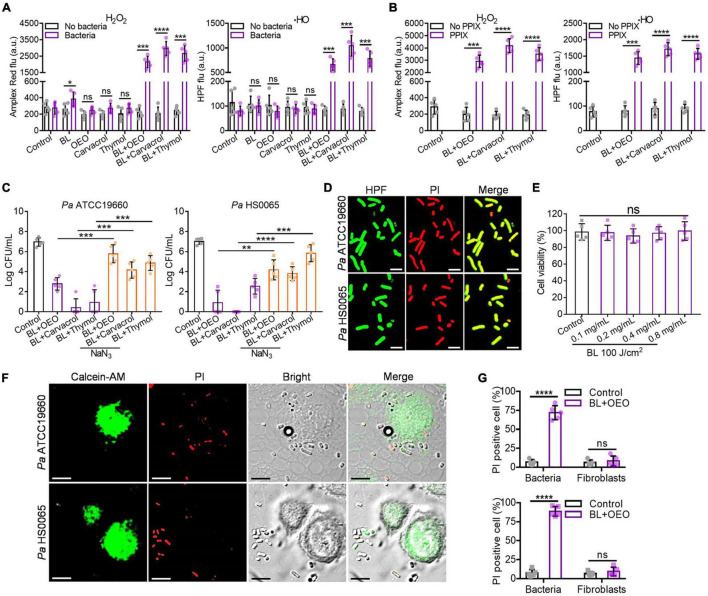
Intracellular ROS are generated specifically in bacteria and play an essential role for the bactericidal action. **(A)** The yields of H_2_O_2_ and HO in the absence or presence of *Pa* HS0065 after corresponding treatments. The dose of OEO, carvacrol, or thymol was the same each at 0.1 mg/ml, and BL was 30 J/cm^2^. **(B)** The generations of H_2_O_2_ and ⋅HO in the absence or presence of 10 μM PPIX under similar treatments as **(A)**. **(C)** Antibacterial efficacy of a combination of BL with OEO, carvacrol, or thymol in a compound dose and fluence as **(A)** against *Pa* ATCC19660 (left) and *Pa* HS0065 (right) in the absence or presence of 10 μM NaN_3_. **(D)** Fluorescence images of *Pa* ATCC19660 and *Pa* HS0065 treated with a duo therapy: 60 J/cm^2^ BL and 0.4 mg/ml OEO for *Pa* ATCC19660 and 30 J/cm^2^ BL and 0.4 mg/ml OEO for *Pa* HS0065. **(E)** The viability of fibroblasts treated by different concentrations of OEO along with 100 J/cm^2^ BL was analyzed by CCK-8 test. **(F)** Fluorescence images of *Pa* ATCC 19660 and *Pa* HS0065 co-cultured with fibroblasts and treated with 50 J/cm^2^ BL and 0.6 mg/ml OEO. PI^+^ cells (red) were dead cells, and calcein-AM^+^ (green) cells were alive cells. Scale bars, 10 μm. **(G)** Co-cultures of PI^+^ bacteria and fibroblasts were counted manually and converted to percentages relative to a total number of the cells. All data are presented as mean ± *SD* of five biological replicates. **(D,F)** Represent as five independent experiments. ****p < 0.0001; ***p < 0.001; **p < 0.01. ns, no significance.

It was found that in comparison with any monotherapy, the yields of H_2_O_2_ and ⋅HO were robustly enhanced by BL combined with OEO, carvacrol, or thymol in a range from 8- to more than 20-fold, which, however, occurred only in the presence of *Pa* HS0065 (Figure 6A, *p* < 0.001). When the bacteria were replaced with PPIX, a photosensitizer commonly found in bacteria including *Pa* (Hamblin et al., 2005; Wang et al., 2016, 2017), similar enhancements of H_2_O_2_ and HO formation were also noticed (Figure 6B). On the other hand, the bactericidal activities were significantly impeded by supplement of NaN_3_, a specific quencher for the singlet oxygen (^1^O_2_), in the parallel study (Figure 6C, *p* < 0.01). Killing of the bacteria resulting mainly from HO was affirmed by perfect merging between HPF^+^ green and PI^+^ red in the bacteria (Figure 6D).

**FIGURE 7:**
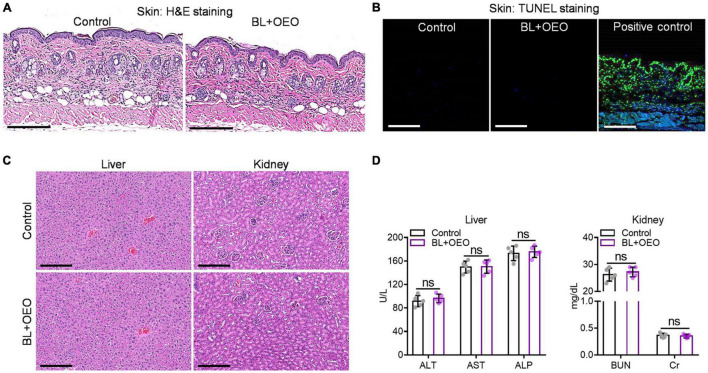
The toxicity of combined BL and OEO *in vivo*. H&E histological examination **(A)** and TUNEL assay **(B)** of the dorsal skin with or without the duo therapy. The dorsal skin was exposed locally to PBS plus sham light (control) or 150 J/cm^2^ BL plus 50 μl of OEO at 50 mg/ml (BL + OEO) daily for 3 consecutive days followed next day by skin biopsy for pathological examinations. Some skin sections were also DNase I treated prior to TUNEL staining as positive control in **(B)**. H&E histological examination **(C)** and related index detections **(D)** of liver and kidney functions from the mice. All data are presented as mean ± *SD* of five biological replicates. Images in **(A–D)** represent as five independent experiments. ns, no significance.

To ascertain that ROS were formed specifically in bacteria and not in mammalian cells, human fibroblasts were treated with a bacteria-lethal dose of BL and OEO. The viability of fibroblasts showed no significant change between control and treated groups (Figure 6E). Besides, fibroblasts were co-cultured with *Pa* ATCC19660 or *Pa* HS0065 and treated with the lethal dose of BL and OEO as above. Most fibroblasts remained viable, evidenced by positive staining of a vital dye calcein-AM and only background levels of PI^+^ cells observed. On the contrary, bacteria were stained chiefly with PI, indicating selective inactivation of bacteria by the combined therapy (Figures 6F,G). The safety was also verified *in vivo*, as manifested by full preservation of dermal tissue structural integrity after irradiation with 150 J/cm^2^ BL in the presence of OEO at 50 mg/ml (BL + OEO) daily for 3 consecutive days (Figure 7A). The doses of BL and OEO were higher than those lethal doses used in the bactericidal assay. Despite a high level of ROS production, we detected no DNA damage or apoptotic cells in the treated tissues, revealed by the TUNEL assay that detected broken DNA or apoptotic cells, consistent with ROS generation primarily within bacteria (Figure 7B). The structure of the liver and kidney remained completely intact (Figure 7C) in the treated group indistinguishable from controls. The index of ALT/AST/ALP and BUN/Cr showed no significant difference between treated and control mice (Figure 7D), in agreement with the histological results (Figures 7A,C). In conclusion, the combined therapy shows harmless to fibroblasts and skin tissues.

## Discussion

Open wound infections of *Pa* can lead to bacterial bloodstream infections, bacteremia, and septicemia (Pont et al., 2020), in association with a high death rate. Should the infection not be kept under check in a timely fashion, the fatality rate can be as high as 45% (Aydın et al., 2018). BL combined with OEO may be a vital alternative to control open wound infections effectively and quickly as it disinfects the wound in < 10 min or about 30 min for biofilms. The modality can be used repeatedly without resistant development. The alternative may be particularly valuable for chronic wound infections. In this regard, patients with chronic, open wounds usually receive a significantly high number of antibiotics, both topically and systemically, and require prolonged hospital stay or frequent doctor visits, during which the patients can acquire additional MDR microbial infections, making even more difficult to treat. Moreover, the MDR bacteria are freely open to the atmosphere and readily spread in the healthcare setting, which presents a great risk to vulnerable patients (such as immunocompromised) in the hospitals. It is thus essential to quickly and efficaciously eliminate bacteria on the wound surface so that nosocomial infections can be minimized, and sepsis prevented in a timely fashion. Apparently, the combined therapy presented in this study represents such a long sought-after approach.

Essential oils are volatile, natural, and fragrant liquids that are extracted from leaves and flowers. More and more these essential oils are found able to kill microbes including MDR ones, such as mustard, thyme, oregano, Chinese cinnamon, etc. Cooperation between two of essential oils were also reported to additively kill bacteria (Ji et al., 2021). However, bactericidal efficacies of these EOs are controversial, mainly because the amount of active ingredients in individual EOs varies with subspecies of the plant/herbs and/or various non-standardized procedures for refinement and fractional distillation of the EOs, giving rise to varying sometime contradict effects. For instance, OEO prepared from *Origanum vulgare* subspecies *hirtum* plants consists of more than 90% of carvacrol, while OEO prepared from *Origanum majorana* contains only 40–50% carvacrol (Daferera et al., 2000; Kokkini et al., 2003; Misharina et al., 2003). The finding that carvacrol and thymol are the major ingredients responsible for the bactericidal activity of OEO, and its synergy with BL offers a unique opportunity to standardize OEO products for their antimicrobial potentials in the basis of the amount of carvacrol and thymol and may also help to identify other EOs for their ability to treat infections. In addition to OEO, carvacrol is also present in the EOs prepared from thyme, pepperwort, and wild bergamot at a concentration ranging from 5 and 75% depending on the plant species and where the plants are grown (De Vincenzi et al., 2004).

The study unravels for the first time that OEO and BL can synergistically eradicate *Pa* owing to the pro-photosensitive characteristics of carvacrol and thymol. Our early investigation showed that BL excited endogenous porphyrins-like molecules, generating singlet oxygen (^1^O_2_) and the superoxide anion radical (O_2_^–^). The ^1^O_2_ and/or O_2_^–^ consequently oxidizes carvacrol and thymol into thymoquinone and thymohydroquinone via endoperoxide (Lu et al., 2021a,b). Thymoquinone and, to a lesser extent, thymohydroquinone act as a photosensitizer generating abundant O_2_^–^ and ^1^O_2_ that in turn oxidize additional carvacrol and thymol, forming more thymoquinone and thymohydroquinone. Thymohydroquinone can be also photo-oxidized into thymoquinone, which is then photo-hydrolyzed into thymohydroquinone while producing H_2_O_2_ and ⋅OH as long as BL is present. These two auto autoxidation cycles are likely to be the underlying mechanism for the synergy between OEO and BL, resembling that of BL plus carvacrol or thymol previously demonstrated (Lu et al., 2021a,b). It is worthwhile to mention that OEO, carvacrol, and thymol are not photosensitizers themselves as they cannot be excited by BL, producing little ROS irrespective of BL’s presence. However, carvacrol, thymol, and OEO function as non-toxic “pro-photosensitizers” that are activated exclusively in bacteria upon BL stimulation (Lu et al., 2021a,b). The super safe profiles of these bacteria-specific pro-photosensitizers are in sharp contrast to conventional photosensitizers used in photodynamic therapy (PDT), wherein the photosensitizers can enter both mammalian cells and bacteria and generate ROS within in response to a specific light, with relatively narrow selectivity.

BL has been broadly employed to treat jaundice in newborns for four decades or acne vulgaris for a decade worldwide (Liebmann et al., 2010; Dai et al., 2012). In contrast to UV, it does not directly interact with DNA and has little risk of carcinogenesis (Liebmann et al., 2010; Dai et al., 2012). It is very safe as long as it does not generate significant heat. We found that the skin temperature remained unaltered up to 200 J/cm^2^ BL exposure and increased 3–5°C after treatment with 300 J/cm^2^. Within the safety range (< 200 J/cm^2^), BL at 405 nm alone has been shown able to sufficiently treat wound infections caused by a number of MDR microbes (Dai et al., 2013a,b; Zhang et al., 2014, 2016; Wang et al., 2016, 2017). The antimicrobial activity of BL relies on the level of non-metallated tetrapyrrole macrocycle production in microbes (Hamblin et al., 2005; Dai et al., 2013a). About 70% of bacterial pathogens generate these porphyrins-like molecules at much higher levels (10–100 ×) than mammalian cells, permitting selective killing of pathogens over mammalian cells (Zhang et al., 2014; Wang et al., 2016, 2017). Another limit of the modality is associated with BL poor tissue penetration, and it is effective mainly on the management of body surface infections.

## Conclusion

In light of a widespread use of OEO for various medical remedy worldwide, this research expands the potential of OEO to disinfection of open wounds or skin infections by its combination with BL. Conceivably, OEO may be combined with sunlight to disinfect the chronic, open wounds in low-income regions, since the intensity of the light used is so low that can be readily obtained *via* sunlight that emits > 50 mW/cm^2^ at a midday of a sunny day or *via* a longer exposure time in a less sunny day. Apart from sunlight, BL LED source is very cheap and can be fabricated as low as one dollar for disinfection. The combined modality is applicable at home to control infections preventing sepsis, which can potentially save many lives in underdeveloped countries.

## Data Availability Statement

The original contributions presented in the study are included in the article/supplementary material, further inquiries can be directed to the corresponding author/s.

## Ethics Statement

The animal study was reviewed and approved by Shanghai Jiao Tong University Animal Study Committee.

## Author Contributions

ML, KW, XL, FW, LW, and SW designed and did all experiments, analyzed the data, and wrote the manuscript. MW supervised the study, analyzed the data, and wrote the manuscript. All authors have read and agreed to the published version of the manuscript.

## Conflict of Interest

The authors declare that the research was conducted in the absence of any commercial or financial relationships that could be construed as a potential conflict of interest.

## Publisher’s Note

All claims expressed in this article are solely those of the authors and do not necessarily represent those of their affiliated organizations, or those of the publisher, the editors and the reviewers. Any product that may be evaluated in this article, or claim that may be made by its manufacturer, is not guaranteed or endorsed by the publisher.
